# Development of an Autophagy Score Signature for Predicting Overall Survival in Papillary Renal Cell Carcinoma

**DOI:** 10.1155/2020/8867019

**Published:** 2020-11-09

**Authors:** Xiang Gu, Xiaojun Chen, Lei Zhu, Wenbo Song, Ali Wang, Junfeng Chu, Tao Wang, Peng Jiang, Yizhi Ge

**Affiliations:** ^1^Department of Oncology, Jiangdu People's Hospital Affiliated to Medical College of Yangzhou University, Yangzhou 225200, China; ^2^Department of Pharmacy, Yizheng People's Hospital, Yizheng 211400, China; ^3^Department of Oncology, Yizheng People's Hospital, Yizheng 211400, China; ^4^Jiangsu Cancer Hospital & Jiangsu Institute of Cancer Research & The Affiliated Cancer Hospital of Nanjing Medical University, China

## Abstract

**Background:**

Autophagy is considered to be closely associated with cancer, functioning as either an anticancer or procancer mechanism depending on the cancer stage. However, the prognostic value of autophagy on papillary renal cell carcinoma (pRCC) remains unclear. In this study, our purpose was to determine the autophagy-related mRNA signature to predict the overall survival of patients with pRCC.

**Materials and Methods:**

A total of 284 patients with pathologic confirmed pRCC in The Cancer Genome Atlas (TCGA) dataset were recruited and included. We choose patients who have smoked less than 15 years but staging 3 or 4 (including nontobacco exposure) vs. more than 15 years but staging 1 or 2. Fourteen differentially expressed mRNAs were found with fold change > 2 and *P* value < 0.001 through *limma* package after making a pair between nontobacco exposure or less than 15 years and tobacco exposure more than 15 years by *matchIt* package.

**Results:**

Six mRNAs were identified to be significantly associated with overall survival. Then, using a risk score based on the signature of these six mRNAs, we divided the patients into low-risk and high-risk groups with significantly different OS. Further multivariate Cox regression analyses revealed that the 6-mRNA signature was independent of age, TNM stage, and tumor type. In the present study, a novel 6-mRNA signature that is useful in survival prediction in pRCC patients was developed. If validated, this mRNA signature might assist in selecting high-risk subpopulation that needs more aggressive therapeutic intervention. The risk score involved in several cancer-related pathways was identified using gene set enrichment analysis.

**Conclusion:**

We initially generated a six autophagy-related genes' signature, which correlates with AJCC N stage, tumor type, and pathological stage and independently predicts OS.

## 1. Introduction

With an estimated 338,000 new cases in 2012, renal cell carcinoma (RCC) accounts for 2.4% of all cancer cases worldwide [1]. According to the WHO classification for tumors of the urinary system, RCC is divided into 5 main histologic subtypes: clear cell (70%-80%), papillary (14%-17%), chromophobe (4%-8%), collecting duct (<1%), and unclassified [2]. Based on the histomorphological characteristics, pRCCs can be subclassified into two distinct subtypes further [3]. Independent studies have demonstrated that type 2 pRCCs (pRCC2) are associated with a worse clinical outcome compared to type 1 pRCCs (pRCC1). While type 2 pRCCs contain multiple molecular subtypes, the type 1 pRCCs are a very homogeneous group. Therefore, to further grasp the prognosis and to develop new biological therapies, cognition of rising biomarkers for pRCC is in an exigent need.

Although the etiology of pRCCs is not clear, pRCCs has been linked to tobacco smoke (TS), exposure to radiation or chemicals, and other risk factors. Studies have shown that ever smoking produced a relative risk for RCC of 1.38, and risk increases were generally greater among men (RR = 1.50) than women (RR = 1.27) [4]. The highest quartiles of combined home/work environmental tobacco smoke exposure among never-smokers, especially with public environmental tobacco smoke exposure, increased RCC risk by 2 to 4 times [5].

Although there are many studies that have shown that the expression of a single gene is associated with tobacco of pRCCs, which includes not only RNA encoding but also many studies on noncoding RNA [6, 7], the current study has no way to clearly explain its overall survival mechanisms. Autophagy is a self-balancing mechanism that can grade long-lived or damaged proteins and organelles to relieve metabolic stress under starvation conditions by recycling intracellular components [8–10]. In cancer cells, autophagy has both tumor-promoting and tumor-suppressive properties, depending on the stage of cancer progression [11]. Autophagy can play a role in cell death in cancer cells that have defects in apoptosis. Autophagy can also promote oncogene-induced senescence or protect the tumor from necrosis and inflammation, thereby promoting tumor growth. Once cancer has formed, autophagy can promote tumor progression by allowing tumor cells to survive stressful conditions [12]. Infinite diffusion is a hallmark of cancer and requires high levels of nutrition and oxygen. Therefore, we conducted this study to further tap the available datasets. By using The Cancer Genome Atlas (TCGA) dataset, we asked if there was a set of mRNA that could distinguish between more aggressive phenotypes and poor survival outcomes. Then, we try to produce a novel signature; the survival of pRCC patients has a better prediction of the results.

## 2. Materials and Methods

### 2.1. Patients and Tissue Samples

The mRNA expression information of TCGA pRCC (KIRP) RNA-sequencing database and the full clinical dataset of TCGA KIRP (up to Aug 11, 2016) are available from The Atlas of Noncoding RNAs in Cancer (TANRIC) (http://bioinformatics.mdanderson.org/main/TANRIC:Overview) [13] and the website of UCSC Xena (http://xena.ucsc.edu/), respectively. The 232 autophagy genes were obtained from the HADb (Human Autophagy Database, http://autoph-agy.lu/clustering/index.html), which provides a list of human autophagy-related genes described in literature [14]. The exclusion criteria were set as follows: (1) samples with clinical data but without TNM stage data (*n* = 3 cases), (2) samples with clinical data but without OS data (*n* = 3 cases), and (3) missing important clinical data, including age and diagnosis subtype (*n* = 2 cases). Overall, 284 patients, which had both mRNA expression data and corresponding clinical information, were included in our study. Moreover, the mRNA expression data for 60 patients in adjacent nontumor tissues were also retrieved.

### 2.2. Statistical and Data Mining Analyses of TCGA KIRP mRNA Profiles

Tumor type, age at initial pathologic diagnosis, and gender were used to ensure the clinicopathological features of the two groups (ratio = 1, caliper = 0.05). Finally, we got 16 vs. 16 patients (no relapse or metastasis vs. relapse or metastasis-related TS) of the matched clinicopathological features of the patients.

We utilized “*limma*” packages to identify differentially expressed autophagy genes among normal and KIRP [15]. The threshold of fold change and *P* values were set as LogFC = log_2_(2) and *P* value < 0.001, respectively. Heat map of differentially expressed mRNAs was plotted with “*pheatmap*” package. The different mRNAs were analyzed in a large sample using two items of logistic regression and single factor Cox proportional hazards regression (median expression as cut-off point) to determine potential mRNA associated with overall survival by “*osgeneral*” package. After the selection of mRNAs, the risk score was calculated based on the sum of mRNA (*i*) expression × coefficient (*i*). The coefficient of each gene was measured by a multivariable Cox regression hazard model with selected autophagy genes. The Kaplan–Meier survival analysis was used to assess the survival distribution between classification groups. The logarithmic rank test was used to assess the statistical significance between stratified survival groups. The area under the ROC curve (AUROC) is calculated from the ROC curve with “*survivalROC*” package. The bilateral *P* value < 0.05 was considered significant. All data above were analyzed using packs in R 2.15.3 and SPSS for Windows, version 22. The work has been reported in line with the REMARK criteria.

## 3. Results

### 3.1. Patient Characteristics

All of the 284 patients included in our study were confirmed by pRCC pathology. The mean age at diagnosis was 61.32 (SD, 11.893), and the mean follow-up was 34.94 months (Table 1). In addition, a total of 60 patients with adjacent nontumor tissue were not involved in our screening for differential expression of mRNAs between pRCC and nontumor tissue. During follow-up, 42 patients eventually died.

### 3.2. Identification of Relapse-Related Autophagy Genes

The expression of mRNA in 16 vs. 16 pRCC tissues (smoking less than 15 years but staging 3 or 4 (including nontobacco exposure) vs. more than 15 years but staging 1 or 2) was involved in this study. 14 differentially expressed mRNAs were screened, with more than Log(FC) = log_2_(2) change; *P* value was <0.001. Of the 14 mRNAs, 10 mRNAs (71.4%) were downregulated and the remaining 4 mRNAs (28.6%) were upregulated (Table S1).

### 3.3. Development of Autophagy Gene-Based Signature

**Figure 1:**
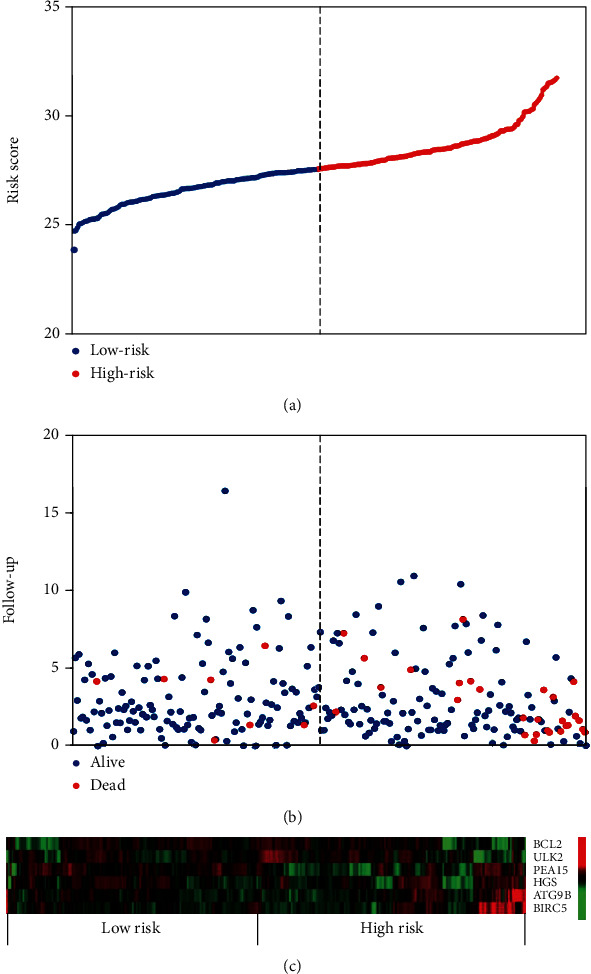
Analysis of 6-mRNA risk score in TCGA patients. The distribution of 6-mRNA risk score, OS status, and mRNA expression profiles were analyzed in TCGA patients (*n* = 284). (a) Six autophagy-related genes' risk score distribution; (b) patient's status and time. The dashed line in the middle divides the patients into the low-risk and high-risk groups. Patients in the high-risk group had higher survival rate (133/142 vs. 109/142, *P* < 0.001) and shorter overall survival times (log rank *P* = 0.001). (c) Heat map of six autophagy-related genes' expression profiles. As the risk score increased, the expression of BCL2 and HGS decreased and the remaining four mRNAs increased. The line represents the autophagy-related genes in the signal, and the column represents the patient.

In order to identify potential mRNAs with prognostic features, different expression mRNAs were further analyzed using the binomial logistic regression and univariate Cox proportional hazards regression, and six mRNAs were found to be significantly associated with overall survival (<0.05). Four of these six mRNAs (ATG9B, BIRC5, HGS, and PEA15) were positively correlated with overall survival, and the remaining two mRNAs (BCL2 and ULK2) were negatively correlated with overall survival. The formula is “risk score = ∑_m=1_^1^mRNA(m)expression × coefficient(n).” According to the risk score, the median risk was taken as the critical value, and the patients were divided into the high-risk group (*n* = 142) and the low-risk group (*n* = 142). Compared with the low-risk group, the high-risk group of patients has poor survival overall (Figure 1). There were significant differences in the distribution of tumor type, pathological stage, lymphonodus status, AJCC T stage, and smoking status, but not in laterality and age (Table 2).

In addition, univariate and multivariate Cox regression analyses were used to test the prognostic impact of 6-mRNA signature on overall survival. As summarized in Table 3, univariate analysis showed that 6-mRNA signature, tumor type, lymphonodus status, AJCC T stage, and pathological stage but not gender, age, laterality, and smoking status were significantly associated with patient overall survival. In a further multivariate analysis, the 6-mRNA signature was still significant for its significance by two-sided log rank test (HR, 4.573; 95% CI, 1.898-11.021), revealing that the 6-mRNA signature was an independent prognostic factor.

**Figure 2:**
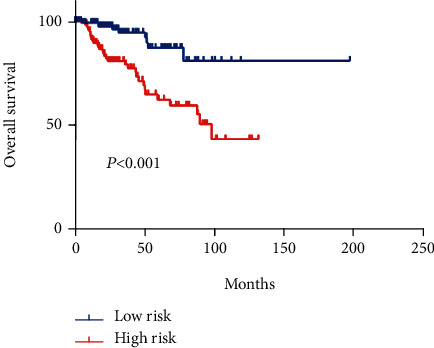
Kaplan–Meier estimation of overall survival in pRCC patients using six autophagy-related genes in TCGA dataset.

**Figure 3:**
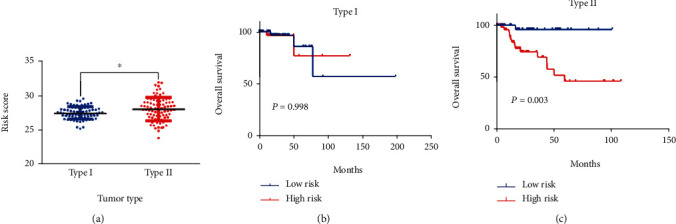
(a) Boxplot of risk score in patients with different tumor types (type 1 vs. type 2). As can be observed, risk score was significantly lower in patients with tumor type (*P* = 0.0105). Kaplan–Meier estimates of overall survival of patients with (b) type 1 and (c) type 2 pRCC in subgroups using the six autophagy-related genes' signature.

Considering that 6-mRNA-based risk scores were not associated with tumor type independently, we further found a positive correlation between them (*P* = 0.001, Figure 2). Patients with type 2 tended to be at high-risk scores. Therefore, we performed a subgroup analysis to determine if the prognostic value of 6-mRNA signature is suitable for all patients, regardless of the tumor type. As shown in Figure 3, we found that patients with type 2 were statistically significant (*P* = 0.003) but there is no statistical significance for patients with type 1 (*P* = 0.998).

Apart from that, we also tested its performance in prediction of relapse-free survival (RFS). Surprisingly, we found a distinct statistical difference between these two groups in 100 months (log rank *P* = 0.0074) (Figure S1). Moreover, in the multivariate Cox regression analysis, the 6-mRNA signature remained a prognostic factor of RFS, independent of AJCC T stage, lymphonodus status, pathological stage, and tumor type (Table S2).

**Figure 4:**
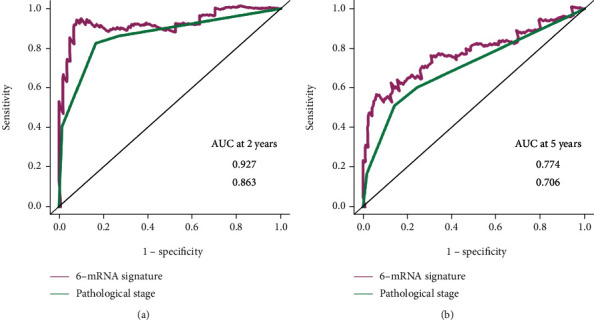
ROC curves of the multivariate logistic regression model with risk score of the six autophagy-related genes' signature or with pathologic stage in prediction of (a) 2-year and (b) 5-year overall survival. As can be observed, the addition of the six autophagy-related genes' signature leads to 6.4% and 6.8% increase of the accuracy in prediction of 2- and 5-year overall survival, respectively.

And then, we performed ROC analyses to compare the predictive accuracy calculated from the multivariate logistic model with or without the 6-mRNA signature. As can be observed, the addition of the 6-mRNA signature leads to 6.4% and 6.8% increase of the accuracy in the prediction of both 2-year and 5-year overall survival (AUROC, 0.927 vs. 0.863; 0.774 vs. 0.706, respectively, Figure 4).

### 3.4. Signaling Pathway Enrichment Analysis Guided by Autophagy Gene Signature

**Figure 5:**
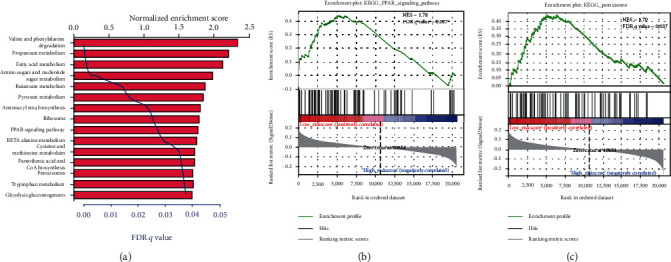
Gene set enrichment analysis (GSEA) of the six autophagy-related genes' signature in TCGA dataset. The significant 15 biological processes and signaling pathway (a). GSEA validated enhanced activity of (b) PPAR signaling pathway and (c) peroxisomes.

We carried out the GSEA to identify the potential associated biological processes and signaling pathway. As displayed in Figure 5, several cancer-related pathways such as PPAR signaling pathway and peroxisomes were enriched in the low-risk group, which revealed that the 6-mRNA signature might be involved in the metabolism.

## 4. Discussion

Papillary renal cell carcinoma is the second most common among RCC. Some new drugs, such as PD-L1 inhibitors, propose novel treatment options and improved prognosis [16]; yet, the heterogeneity of tumors necessitates the exploration of individualized treatments and prognostic biomarkers. Autophagy-related RNAs are an intensive research topic in molecular biology with numerous studies; however, the oncologic value of mRNA is yet unclear in the clinical setting. Thus, the current study focused on the clinical application of mRNA in pRCC and explored the underlying complex biological function involved in various cancer types. Moreover, some mRNAs such as SIRT6 [17], FTY720 [18], AMBRA1 [19], ULK1, and LC3B [20] were related to worse characteristics like higher TNM stage, progression, metastasis, or poorer survival outcomes. The combination of candidate mRNAs not only improves accuracy but also reduces this difference. In recent years, signature [21–24] based on RNA-sequencing analysis has been developed to identify subgroups that have a more aggressive phenotype or poor survival outcome in several cancer types.

However, most construction of signature failed to describe the clinical features and was unable to relate to the clinical needs. The selection of signature was detected between the tumor tissue and adjacent tissues, which cannot really reflect the clinical features. In our study, we were the first to demonstrate that some nontobacco smoker and smokers who smoke for shorter periods were detected to have recurrence or metastasis in a short term. However, smokers for longer periods may have a good prognosis. Between these two groups, we found 14 differentially expressed mRNAs. Among these mRNAs and through excavating the 284 pRCC patients' RNA-sequencing data, we have identified a 6-mRNA signature that was correlated with tumor type associated with poor survival. The further multivariate analyses revealed that the 6-mRNA signature was an independent predictor of pRCC patients' RFS and OS.

To test the independence of our 6-mRNA signature in predicting overall survival, we conducted a multivariate Cox regression analysis including tumor type, lymphonodus status, AJCC T stage, and pathological stage as covariates. pRCC patients with type 2 are associated with pRCC-related survival prognosis. Similarly, other parameters of malignancy including AJCC T stage and lymphonodus status also have a prognostic impact on patient survival. In our univariate analysis, all of these covariates showed significant correlation with overall survival. However, in the multivariate analysis, one of the five covariates mentioned lost the prediction of survival. However, the risk score for 6-mRNA signature maintains the prognostic impact on overall survival. Therefore, we can conclude that our new signature is an independent prognostic factor for overall survival. In addition, we also found a positive correlation between the risk score and the AJCC T stage. As shown in Figure 3, the tumor type patients tended to be high risk scores (*P* = 0.0105). Subgroup analyses based on tumor type further progressed. Surprisingly, the statistical significance of survival curve was among patients with only type 2. We can conclude that our signature is suitable for patients with type 2.

In addition, ROC analysis showed that our 6-mRNA signature AUROC was 0.927 in prediction of 2-year overall survival and 0.774 in prediction of 5-year overall survival, more than pathological stage. Currently, pathological stage will be an element of the risk score and has been shown to correlate with patient survival.

As for the characteristics of the six mRNAs, overexpression of BCL2 and ULK2 correlated with a lower survival rate of overall survivors, while the other four mRNAs (ATG9B, BIRC5, HGS, and PEA15) were significantly higher in the high-risk group relative to the low-risk group. At present, a number of studies reported and revealed that the proliferation of pRCC cell lines may be regulated by specific mRNAs. For example, upregulated mRNA BCL2 in pRCC tissues was shown to affect the proliferation of pRCC cell lines through regulation of CD24 and CD133 [25]. In order to better understand these mRNAs in pRCC, additional functional studies may be worthwhile.

Several limitations should be considered. Firstly, partial mRNAs that we include were not annotated in the dataset, but we still included in this study. But the six selected prognostic mRNAs can represent all the mRNA candidates that are potentially correlated with overall survival of pRCC. On the other side, we lack a mechanism for investigating the prognostic value of mRNA in pRCC. In addition, the specific role of 6 mRNAs on pRCC phenotype is unclear. Finally, our findings are summarized in a published dataset without a prospective test in clinical trials. So a larger sample size makes our findings more convincing. To date, several gene set based-signatures have been developed in hopes of providing prognostic and predictive value in renal cancer. However, before the signature can be applied as a clinical-grade assay, further steps are needed to be determined including establishment of a standard procedure of sample preprocessing and stable next-generation sequencing platform and validation in independent cohorts of patients with full clinical annotation available.

## 5. Conclusion

In conclusion, we developed a six-autophagy-related mRNA signature composed of various regulation mRNA that effectively classify pRCC patients into low-risk and high-risk groups. The clinical significance of these six mRNAs deserves further study.

## Figures and Tables

**Table 1:** Clinicopathological characteristics of the patients with papillary renal cell carcinoma in TCGA.

Characteristics	TCGA cohort (*N* = 246)	
	*N*	%
Age, median (range)	61.32	28–88
Gender		
Male	208	73.2
Female	76	26.8
Tumor type		
Type I	75	26.3
Type II	83	29.1
Unknown	127	44.6
pT stage		
T1	191	67.3
T2	33	10.7
T3	57	17.6
T4	2	0.7
pN stage		
N0	50	17.6
N1	23	8.1
N2	4	1.4
Nx	207	72.9
Pathologic M		
M0	93	32.7
M1	9	3.2
Mx	182	64.1
Pathological stage		
Stage I	189	66.5
Stage II	28	9.9
Stage III	51	18.0
Stage IV	16	5.6
Laterality		
Bilateral	2	0.7
Left	157	55.3
Right	124	43.7
Smoking status		
Lifelong nonsmoker	115	40.5
Current smoker	35	12.3
Current reformed smoker for >15 years	45	15.8
Current reformed smoker for ≤15 years	38	13.4
Unknown	40	14.1

**Table 2:** Correlations analysis between signature-based risk group and clinicopathological characteristics.

Variable	Low risk	High risk	*χ* ^2^	*P* value
Age of diagnosis			2.167	0.141
≤65	83	95		
>65	59	47		
AJCC T stage			**19.018**	**<0.001**
T1+T2	127	97		
T3+T4	15	45		
Lymphonodus status			**5.683**	**0.017**
Negative	17	22		
Positive	7	31		
Tumor type			**7.335**	**0.007**
Type I	46	29		
Type II	33	50		
Pathological stage			**21.272**	**<0.001**
Stage I+stage II	125	92		
Stage III+stage IV	17	50		
Laterality			3.494	0.062
Left	71	86		
Right	70	54		
Smoking status			**3.989**	**0.046**
Nonsmoker and smoker for ≤15 years	67	85		
Smoker for >15 years	28	18		

**Table 3:** Univariate and multivariate Cox regression analyses of autophagy signature in predicting OS.

Variable	Univariate analysis			Multivariate analysis		
	HR	95% CI	*P* value	HR	95% CI	*P* value
6-RNA signature	2.715	2.135-3.454	**<0.001**	4.573	1.898-11.021	**0.001**
Age	1.002	0.976–1.029	0.885			
Gender	1.632	0.843–3.160	0.146			
Tumor type	2.849	1.041-7.798	**0.042**	0.081	0.007–0.912	**0.042**
AJCC T stage (T1-2 vs. T3-4)	5.020	2.737–9.207	**<0.001**	1.834	0.339–9.919	0.182
N stage	5.211	2.149–12.636	**<0.001**	0.975	0.161–5.911	0.004
Pathological stage (I-II vs. III-IV)	2.051	1.393–3.201	**<0.001**	2.154	0.073–63.570	0.004
Laterality (left vs. right)	0.760	0.403–1.431	0.395			
Smoking status	0.352	0.122–1.015	0.053			

## Data Availability

Source data and reagents are available from the corresponding author upon reasonable request.

## Supplementary Materials

Supplementary 1 Figure S1: Kaplan–Meier estimation of RFS in pRCC patients using six autophagy-related genes in TCGA database.

Supplementary 2 Table S1: the detailed information of selected mRNAs.

Supplementary 3 Table S2: univariate and multivariate Cox regression analyses of the autophagy signature in predicting RFS.
