# Supplementary figures and images for: Development of an Autophagy Score Signature for Predicting Overall Survival in Papillary Renal Cell Carcinoma
Source: Dis Markers. 2020 Nov 9;2020:8867019. doi: 10.1155/2020/8867019 (PMC7684156; doi:10.1155/2020/8867019)

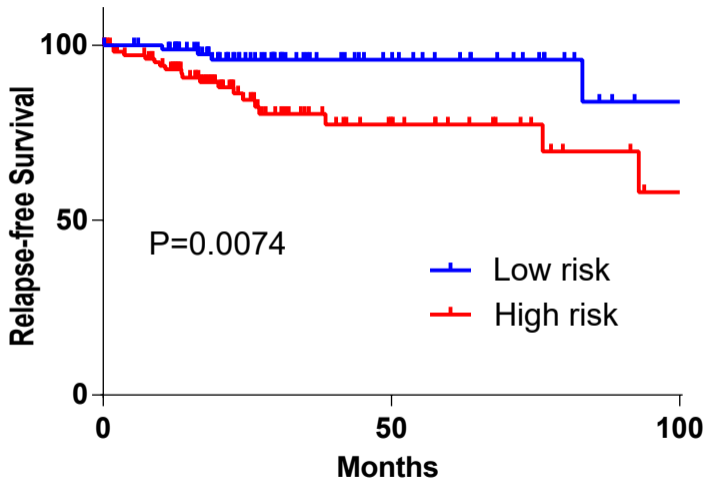

Figure S1

Supplement: Supplementary 1 — Figure S1: Kaplan–Meier estimation of RFS in pRCC patients using six autophagy-related genes in TCGA database. [file 8867019.f1.pdf]
