# Supplementary material for: Development of an Autophagy Score Signature for Predicting Overall Survival in Papillary Renal Cell Carcinoma
Source: Dis Markers. 2020 Nov 9;2020:8867019. doi: 10.1155/2020/8867019 (PMC7684156; doi:10.1155/2020/8867019)
Supplement: Supplementary 2 — Table S1: the detailed information of selected mRNAs. [file 8867019.f2.docx]

**Table S1.** The detailed information of selected mRNAs

|  | | | |
| --- | --- | --- | --- |
| Gensymbol | logFC | P.Value | adj.P.Val |
| WDFY3 | 9.435206 | 2.99E-05 | 0.002747 |
| BIRC5 | -16.962 | 3.54E-05 | 0.002747 |
| IFNG | -8.28004 | 0.000396 | 0.023048 |
| BCL2 | 11.69159 | 0.000765 | 0.035654 |
| C12orf44 | -7.44076 | 0.001259 | 0.048908 |
| SPNS1 | -5.84039 | 0.001977 | 0.05615 |
| HGS | -3.08159 | 0.00204 | 0.05615 |
| TP63 | -6.07755 | 0.002169 | 0.05615 |
| PEA15 | -3.49239 | 0.003205 | 0.074676 |
| ULK2 | 12.12594 | 0.004319 | 0.084056 |
| ATIC | -6.39439 | 0.004358 | 0.084056 |
| WIPI1 | 6.912729 | 0.00469 | 0.084056 |
| TMEM74 | -3.38506 | 0.007189 | 0.119641 |
| ATG9B | -9.0016 | 0.00965 | 0.149895 |
