# Supplementary material for: Development of an Autophagy Score Signature for Predicting Overall Survival in Papillary Renal Cell Carcinoma
Source: Dis Markers. 2020 Nov 9;2020:8867019. doi: 10.1155/2020/8867019 (PMC7684156; doi:10.1155/2020/8867019)
Supplement: Supplementary 3 — Table S2: univariate and multivariate Cox regression analyses of the autophagy signature in predicting RFS. [file 8867019.f3.docx]

**Table S2.** Univariate and multivariate Cox regression analyses of the autophagy signature in predicting RFS.

|  | | |  |  | | |  | |  | |  |
| --- | --- | --- | --- | --- | --- | --- | --- | --- | --- | --- | --- |
| Variable | Univariate analysis | | | | | Multivariate analysis | | | | | |
|  | HR | 95% CI | | | P value | HR | | 95% CI | | P value | |
| Risk score (high vs. low) | 2.395 | 1.744-3.290 | | | **<0.001** | 2.511 | | 1.392-4.531 | | **0.002** | |
| Age | 0.996 | 0.960 – 1.033 | | | 0.821 |  | |  | |  | |
| Gender | 2.997 | 1.253 – 7.168 | | | **0.014** |  | |  | |  | |
| Tumor type | 2.562 | 0.662 – 9.918 | | | 0.173 | 0.365 | | 0.045 – 2.977 | | 0.348 | |
| AJCC T stage (T1-2 vs. T3-4) | 4.731 | 1.852 – 12.088 | | | **0.001** | 1.392 | | 0.123 – 15.742 | | 0.182 | |
| N stage | 4.445 | 0.963 – 20.513 | | | 0.056 |  | |  | |  | |
| Pathological stage(I-II vs.III- IV) | 5.175 | 2.196 – 12.196 | | | **<0.001** | 1.392 | | 0.304 – 54.730 | | 0.289 | |
| Laterality(Left vs Right) | 0.659 | 0.265 – 1.640 | | | 0.370 |  | |  | |  | |
| Smoking status | 1.103 | 0345 – 3.523 | | | 0.869 |  | |  | |  | |
